# Prophylactic Supraclavicular Lymphadenectomy Does Not Improve Prognosis in Upper and Middle Thoracic Esophageal Squamous Cell Carcinoma: A Retrospective Single-Center Study

**DOI:** 10.3390/medicina62040625

**Published:** 2026-03-25

**Authors:** Tomotake Ariyoshi, Koji Otsuka, Masahiro Kohmoto, Akira Saito, Kentaro Motegi, Takeshi Yamashita, Satoru Goto, Masahiko Murakami, Takeshi Aoki

**Affiliations:** 1 Division of Gastroenterological and General Surgery, Department of Surgery, School of Medicine, Showa Medical University, Tokyo 142-8555, Japantyamashita@med.showa-u.ac.jp (T.Y.);; 2 Esophageal Cancer Center, Showa Medical University Hospital, Tokyo 142-8666, Japan; 3 Digestive Disease Center, Showa Medical University Koto Toyosu Hospital, Tokyo 135-8577, Japan

**Keywords:** esophagectomy, supraclavicular lymph node dissection, minimally invasive surgery, squamous cell carcinoma, upper or middle thoracic esophageal cancer

## Abstract

*Background and Objectives*: The benefits of prophylactic supraclavicular lymph node dissection for esophageal squamous cell carcinoma (ESCC) remain controversial. This study investigated whether prophylactic supraclavicular (cervical) lymphadenectomy improves the long-term outcomes of patients with upper or middle thoracic esophageal squamous cell carcinoma. *Materials and Methods*: This retrospective, single-center study included 290 patients who underwent thoracoscopic esophagectomy between January 2010 and December 2017. Patients treated with two-field lymphadenectomy (2FL) were compared with those who underwent prophylactic three-field lymphadenectomy (p3FL) after propensity score matching based on age, tumor location, clinical T and N stage, and preoperative treatment. The primary outcome was overall survival (OS), and secondary outcomes included postoperative complications and recurrence patterns. In a secondary analysis, the long-term outcomes were assessed in patients with solitary postoperative cervical (supraclavicular) lymph node recurrence in the 2FL group. *Results*: In the overall cohort, statistically significant differences were observed between the groups with respect to age, tumor location (*p* = 0.0002), cT and cN stages (*p* < 0.0001 and *p* < 0.0001), preoperative treatment (*p* = 0.02). No significant differences were observed between groups regarding age, organ for reconstruction, or postoperative complications. After propensity score matching, no significant differences were observed between the 2FL and p3FL groups in terms of overall survival or postoperative complications. Six patients (4.4%) in the p3FL group had pathologically confirmed supraclavicular lymph node metastasis, whereas four patients (2.6%) in the 2FL group developed solitary postoperative cervical lymph node recurrence. Patients with isolated cervical recurrence achieved favorable long-term survival following additional treatment. *Conclusions*: Prophylactic cervical lymphadenectomy did not improve the survival of patients with upper or middle thoracic esophageal squamous cell carcinoma. Given the low incidence of isolated cervical lymph node recurrence and the favorable outcomes achievable with additional treatment, routine prophylactic supraclavicular dissection appears unnecessary when two-field lymphadenectomy is feasible.

## 1. Introduction

Esophageal squamous cell carcinoma (ESCC) remains one of the most aggressive malignancies of the gastrointestinal tract and continues to represent a major oncologic challenge worldwide. In East Asian countries, particularly Japan, squamous cell carcinoma accounts for the majority of esophageal cancers and demonstrates a distinct pattern of lymphatic metastasis characterized by extensive longitudinal spread along the recurrent laryngeal nerve chains toward the cervical and upper mediastinal lymphatic basins. This anatomical characteristic historically provided the foundation for the development of three-field lymphadenectomy, which includes cervical, mediastinal, and abdominal nodal dissection.

The concept of extended lymphadenectomy in thoracic esophageal cancer was popularized in Japan during the late twentieth century. Early reports suggested that systematic cervical lymph node dissection could improve locoregional control and potentially enhance survival outcomes [1,2]. Consequently, prophylactic supraclavicular lymph node dissection became widely accepted as a standard surgical approach for upper and middle thoracic squamous cell carcinoma in Japan [3,4]. However, this approach has not been universally adopted internationally.

According to the eighth edition of the TNM classification, supraclavicular lymph nodes are categorized as distant metastasis [5,6]. This classification implies systemic disease and has contributed to more conservative surgical approaches in Western countries. In contrast, the Japanese Classification of Esophageal Cancer (12th edition) considers these nodes regional and potentially amenable to therapeutic dissection [7,8]. This discrepancy reflects a fundamental difference in oncologic philosophy regarding whether cervical lymph node involvement represents locoregional spread or systemic dissemination.

In recent years, treatment strategies for ESCC have evolved considerably. Intensified neoadjuvant chemotherapy regimens, including docetaxel-based combinations and chemoradiotherapy protocols have significantly improved systemic disease control [9,10]. Furthermore, the introduction of immune checkpoint inhibitors may also contribute to improving the prognosis of esophageal cancer in the future. As multimodal therapy becomes increasingly effective, the incremental survival benefit derived from extended prophylactic lymphadenectomy may diminish.

Several studies have examined this procedure across all thoracic esophageal cancers [11,12]. Although this dissection is standard in Japan for squamous cell carcinoma of the upper or middle thoracic esophagus, it is often omitted for lower thoracic esophageal cancer.

The present study therefore aimed to evaluate, using a propensity score–matched analysis, whether prophylactic supraclavicular lymphadenectomy improves overall survival in patients with upper or middle thoracic ESCC undergoing thoracoscopic esophagectomy.

## 2. Materials and Methods

Between January 2010 and December 2017, 643 patients underwent thoracoscopic esophagectomy for esophageal cancer at our institution. The inclusion criteria were as follows: (i) ESCC without clinical evidence of cervical lymph node metastasis; (ii) tumor located in the upper thoracic (Ut) or middle thoracic (Mt) esophagus; (iii) clinical stage I–IVa (TNM classification, 8th edition); (iv) age ≤ 80 years; and (v) radical thoracoscopic esophagectomy (McKeown procedure). Patients with residual tumors (R1 or R2) and those with synchronous or metachronous cancers in other organs that could affect prognosis were excluded. Ultimately, 290 patients were enrolled. After applying these criteria, 290 patients were eligible for analysis.

**Figure 1:**
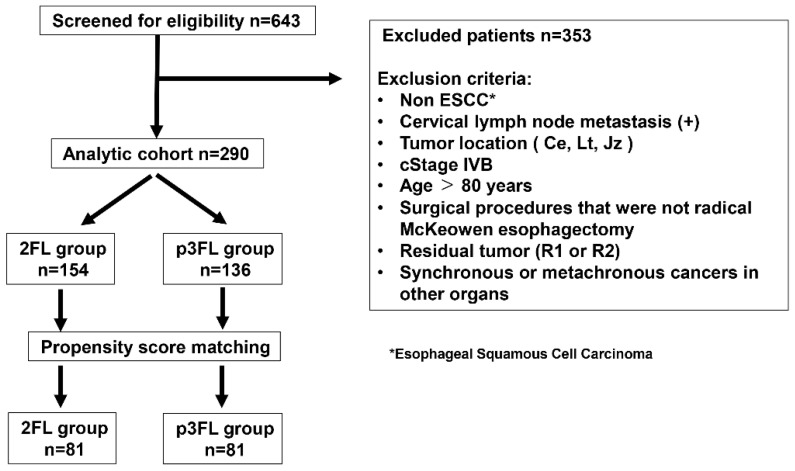
Patient registration criteria in this study.

Patients were divided into two groups according to the extent of lymphadenectomy performed. Those who underwent mediastinal and abdominal lymph node dissection without cervical dissection were classified as the two-field lymphadenectomy (2FL) group. Those who additionally underwent bilateral supraclavicular lymph node dissection were classified as the prophylactic three-field lymphadenectomy (p3FL) group (Figure 1).

### 2.1. Preoperative Assessment

All patients underwent comprehensive preoperative evaluation including upper gastrointestinal endoscopy with biopsy, contrast-enhanced computed tomography (CT) of the neck, chest, and abdomen, and cervical ultrasonography. Positron emission tomography was performed selectively based on clinical indications. As a general rule, a cervical lymph node with a short-axis diameter ≥ 6 mm on CT was considered metastatic. This threshold was based on a study evaluating the diagnosis of preoperative metastasis using CT for cervical paraesophageal and thoracic paratracheal lymph nodes, as described in the Japanese Classification of Esophageal Cancer (12th edition) [8]. In addition, reports have suggested that supraclavicular lymph nodes with a short-axis diameter of ≥6 mm are diagnosed as metastatic [13]. The final clinical stage was determined based on a comprehensive assessment of all examination results.

### 2.2. Neoadjuvant Therapy

For patients with cT2–4 disease or clinically positive lymph node metastasis, neoadjuvant chemotherapy with cisplatin and 5FU (CF), including a low-dose regimen with nedaplatin or docetaxel, cisplatin, and 5FU (DCF), was the standard treatment at our institution. Radiotherapy (40–60 Gy) was administered in combination with CF for locally advanced esophageal cancer.

### 2.3. Surgical Procedure

All patients underwent thoracoscopic subtotal esophagectomy in the left lateral decubitus position with two-field or three-field lymph node dissection [14,15]. Robotic surgery was not performed in this study.

The abdominal procedure was performed using hand-assisted laparoscopic surgery. Reconstruction was based on the creation of a subtotal gastric conduit; if gastric reconstruction was not feasible, colonic interposition was performed.

Bilateral supraclavicular lymph node dissection was performed as specified in the Japanese Classification of Esophageal Cancer (12th edition) [7,8]. The dissection boundaries were defined as follows: medial, the edge of the carotid sheath; cranial, a level above the cricoid cartilage; caudal, the upper edge of the subclavian vein; and lateral, the supraclavicular nerve.

### 2.4. Outcome Measures

The primary endpoint was overall survival. Secondary endpoints included recurrence patterns and postoperative complications. Recurrence was categorized as local, regional lymph node, cervical lymph node, distant lymph node, other organ metastasis, or mixed recurrence. In addition, detailed analysis was performed for patients who developed isolated cervical lymph node recurrence after two-field lymphadenectomy.

### 2.5. Statistical Analysis

Clinical characteristics were compared between the 2FL and p3FL groups using Fisher’s exact test and the Mann–Whitney U test. The 2FL and p3FL groups were matched according to age, tumor location, cT and cN stages, and preoperative treatment using propensity score matching (PSM). PSM was performed using 1:1 nearest-neighbor matching with a caliper width of 0.20 standard deviations of the logit of the propensity score. Survival curves were estimated using the Kaplan–Meier method and compared using the log-rank test. All analyses were performed using JMP^®^ 17 (SAS Institute Inc., Cary, NC, USA), and EZR (Jichi Medical University, Tochigi, Japan), which is a graphical user interface for R (The R Foundation for Statistical Computing, Vienna, Austria). A *p* value of <0.05 was considered statistically significant.

## 3. Results

A total of 290 patients were included in the study: 154 in the 2FL group and 136 in the p3FL group. In the overall cohort, statistically significant differences were observed between the groups with respect to tumor location, cT and cN stages, preoperative treatment (Table 1).

**Figure 2:**
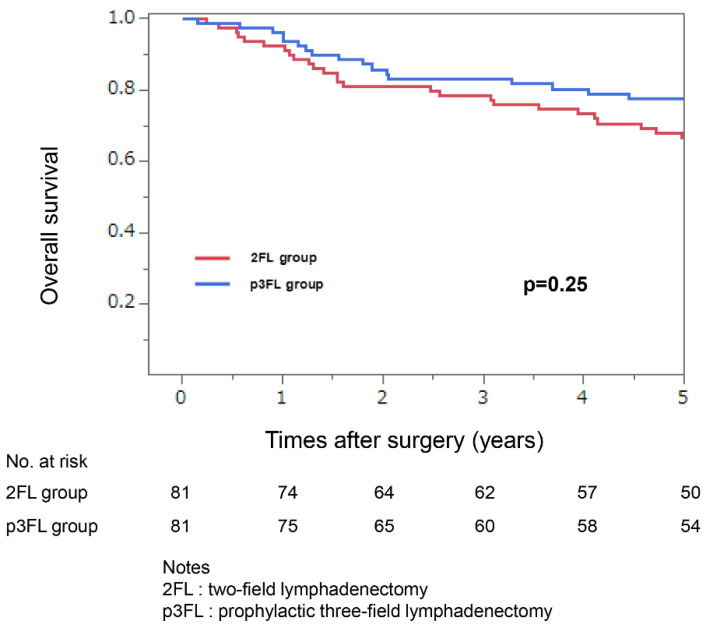
Overall survival in the two-field lymphadenectomy (2FL) and prophylactic three-field lymphadenectomy (p3FL) groups.

PSM was performed between the 2FL and p3FL groups according to age, tumor location, cT and cN stages, and preoperative treatment. The characteristics of the matched cohort are summarized in Table 1. After PSM, no statistically significant differences in patient characteristics were observed between the two groups. No statistically significant difference in overall survival was observed between the groups (Figure 2). The 5-year survival rates in the 2FL and p3FL groups were 66.7% and 77.5%, respectively (*p* = 0.25).

Six patients (4.4%) in the p3FL group had pathologically confirmed cervical lymph node metastasis. In contrast, 4 patients (2.6%) in the 2FL group developed isolated postoperative recurrence in the cervical lymph nodes.

The pattern of postoperative recurrence in the whole cohort is shown in Table 2. Postoperative recurrence occurred in 29 patients (18.8%) in the 2FL group and 50 patients (36.8%) in the p3FL group. Analysis of recurrence patterns revealed Isolated cervical lymph node recurrence was also observed in the p3FL group. In addition to the overall frequency of recurrence, the distribution of recurrence patterns was also examined. Regional lymph node recurrence was more frequently observed in the p3FL group than in the 2FL group; however, this difference was largely attributable to mediastinal or abdominal lymph node recurrence rather than cervical lymph node recurrence. Importantly, the incidence of isolated cervical lymph node recurrence remained low in both groups. These findings suggest that cervical lymph node metastasis may represent only a limited component of the overall recurrence pattern following curative esophagectomy for thoracic ESCC.

Furthermore, we investigated the long-term prognosis of patients with isolated cervical lymph node recurrence after surgery in the 2FL group. The results are shown in Table 3. The median follow-up period was 1744 days (range, 890–2864 days). Importantly, patients who developed isolated cervical recurrence achieved prolonged survival following salvage treatment. One patient survived more than seven years after recurrence, suggesting that isolated cervical recurrence may represent a potentially controllable oligometastatic state.

## 4. Discussion

This study investigated whether prophylactic supraclavicular lymph node dissection confers a survival benefit to patients with upper or middle thoracic ESCC undergoing thoracoscopic esophagectomy. In this propensity score–matched cohort, prophylactic cervical lymphadenectomy was not associated with improved overall survival, and no clear difference in recurrence patterns was observed between the groups. These findings suggest that routine cervical dissection may not be oncologically justified in this specific population when two-field lymphadenectomy is adequately performed.

The role of cervical lymph node dissection in thoracic ESCC has long been debated. In Japan, three-field lymphadenectomy has historically been considered the standard treatment because of the relatively high incidence of cervical lymphatic spread, particularly along the bilateral recurrent laryngeal nerves [1,2,3,4]. This surgical philosophy is rooted in detailed anatomical studies demonstrating longitudinal lymphatic drainage from the upper and middle thoracic esophagus toward the cervical region. The concept of three-field dissection was developed in an era when effective systemic therapies were limited and surgical radicality was considered the primary strategy for achieving oncologic control. In that context, removal of cervical lymph nodes was thought to eliminate potential micrometastatic disease and improve long-term outcomes. In contrast, according to the TNM classification (8th edition), supraclavicular lymph nodes are classified as distant metastases, and routine cervical dissection is not universally adopted in Western countries. This discrepancy reflects fundamental differences in the conceptualization of cervical lymph node metastasis, a regional disease amenable to surgical control versus systemic spread. Therefore, contemporary data are needed to clarify whether extensive cervical dissection provides meaningful prognostic benefits in the era of multimodal therapy.

Although previous randomized trials have reported no survival advantage with prophylactic cervical dissection [11,12,16,17], our findings are consistent with these reports, including two RCTs. In one RCT, eligible patients underwent right thoracotomy, and the tumors were located in the middle or lower thoracic esophagus [16]. In another RCT, minimally invasive esophagectomy was performed, and tumors were distributed throughout the thoracic esophagus, including adenocarcinomas [17]. The present study focused exclusively on squamous cell carcinoma of the upper and middle thoracic esophagus, which are tumor sites likely to involve cervical lymphatic spread. Despite this targeted analysis, no survival benefit was observed, reinforcing the limited oncologic value of routine prophylactic cervical dissection, even in anatomically high-risk tumors.

Accurate preoperative identification of cervical lymph node metastasis is critically important when determining the appropriate extent of lymphadenectomy. In the present study, the rate of pathologically confirmed cervical lymph node metastasis in the p3FL group was 4.4%, while the incidence of isolated cervical lymph node recurrence in the 2FL group was 2.6%. These relatively low rates suggest that the accuracy of preoperative imaging for detecting cervical lymph node metastasis was reasonably maintained in our cohort. Nevertheless, because PET-CT was not routinely performed during the study period, the possibility of occult cervical lymph node metastasis cannot be completely excluded.

Recent advances in imaging modalities may further contribute to improving the preoperative detection of cervical lymph node metastasis. High-resolution contrast-enhanced CT, cervical ultrasonography, and PET-CT have improved the sensitivity of detecting small metastatic lymph nodes. Although PET-CT was not routinely performed during the present study period, its wider adoption in current clinical practice may further reduce the risk of overlooking clinically significant cervical lymph node metastasis. Improved preoperative staging may allow surgeons to reserve cervical lymph node dissection for patients with radiologically suspicious nodes rather than performing prophylactic dissection in all patients.

An important observation in this study was the low incidence (2.6%) of isolated cervical lymph node recurrence after two-field lymphadenectomy. Furthermore, patients with solitary cervical recurrence achieved favorable long-term survival with salvage chemoradiotherapy or radiotherapy. Previous studies have reported good outcomes with salvage surgical lymphadenectomy in selected cases [18,19,20,21,22]; however, the present data suggest that effective nonsurgical salvage treatment may also provide acceptable oncologic control. These findings support the concept that isolated cervical lymph node recurrence may represent an oligometastatic state rather than inevitable systemic dissemination. If so, selective salvage strategies may be more appropriate than routine prophylactic surgery for all patients.

Potential morbidity associated with prophylactic supraclavicular lymph node dissection should also be considered. Although no statistically significant increase in major complications was observed in the matched cohort, prior reports have demonstrated associations between cervical dissection and recurrent laryngeal nerve injury, impaired swallowing, and respiratory complications [23,24,25]. Even modest increases in postoperative dysfunction can meaningfully affect the quality of life in long-term survivors. When the oncologic benefit is uncertain or minimal, avoiding additional surgical trauma is an important consideration.

The clinical implications of these findings are particularly relevant in the context of modern multimodal treatment. Advances in neoadjuvant chemotherapy (including DCF), chemoradiotherapy, and the introduction of immune checkpoint inhibitors, such as adjuvant nivolumab, have significantly improved systemic and locoregional tumor control [26]. As systemic therapy becomes more effective, the incremental benefit of extensive prophylactic lymphadenectomy may diminish. Another consideration is the potential need for more selective indications for cervical lymph node dissection. Although the overall incidence of cervical lymph node metastasis was low in the present cohort, certain subgroups of patients may still benefit from more extensive lymphadenectomy. In such situations, individualized surgical strategies tailored to the predicted pattern of lymphatic dissemination may be more appropriate than a uniform approach for all patients. Future studies incorporating refined risk stratification, advanced imaging modalities, and molecular or biological markers may help identify patients who could derive meaningful benefit from cervical lymph node dissection while avoiding unnecessary surgical morbidity in low-risk populations. In this evolving therapeutic landscape, surgical strategies should be reassessed to balance oncologic radicality with functional preservation.

Another perspective to consider is the evolving role of surgery in the multidisciplinary treatment of esophageal cancer. Historically, extensive lymphadenectomy was considered essential to achieve adequate oncologic clearance. However, the increasing effectiveness of systemic therapy and perioperative treatment strategies has gradually shifted the balance between surgical radicality and treatment-related morbidity. In this context, less invasive surgical strategies that maintain oncologic safety while minimizing postoperative dysfunction are gaining increasing attention. Selective lymphadenectomy guided by accurate staging and individualized risk assessment may represent a more appropriate approach in the modern era.

This study has several limitations. Its retrospective, single-institution design may have introduced selection bias. Although propensity score matching was employed to reduce confounding, residual unmeasured variables cannot be excluded. Additionally, neoadjuvant treatment regimens were not uniform throughout the study period. However, the relatively large sample size, restriction to ESCC, and limitation to upper and middle thoracic tumor locations enhance the reliability of the findings. Future prospective multicenter studies, particularly those incorporating standardized multimodal therapy protocols, are warranted to further clarify the role of prophylactic supraclavicular lymphadenectomy.

## 5. Conclusions

In patients with upper or middle thoracic ESCC undergoing thoracoscopic esophagectomy, prophylactic supraclavicular lymph node dissection was not associated with improved long-term survival. The incidence of isolated cervical lymph node recurrence after two-field lymphadenectomy was low, and favorable outcomes were achieved with salvage treatment. These findings suggest that routine prophylactic cervical dissection should not be considered mandatory when a two-field lymphadenectomy is feasible and clinically adequate. An individualized lymphadenectomy strategy, prioritizing precise mediastinal dissection and reserving cervical intervention for clinically suspected or recurrent disease, may provide a more balanced approach that optimizes oncologic control while minimizing surgical morbidity. In the era of increasingly effective multimodal therapy, including neoadjuvant chemotherapy, chemoradiotherapy, and immunotherapy, the role of extensive prophylactic lymphadenectomy warrants continued reassessment. Well-designed prospective multicenter trials are necessary to define the optimal extent of lymph node dissection in the contemporary management of thoracic esophageal squamous cell carcinoma.

## Figures and Tables

**Table 1:** Characteristics of the patients in the entire cohort and matched cohort after propensity score matching.

	Whole Cohort			Matched Cohort			
Variable	2FL (*n* = 154)	p3FL (*n* = 136)	*p*-value	2FL (*n* = 81)	p3FL *(n* = 81)	*p*-value	SMD
Age	69 (47–80)	64 (39–80)	*p* < 0.0001	65 (47–80)	67 (50–80)	*p* = 1.00	0.117
Male/Female	130/24	109/27	*p* = 0.34	67/14	67/14	*p* = 1.00	<0.001
Location of tumor (Ut/Mt)	12/142	32/104	*p* = 0.0002	11/70	12/69	*p* = 1.00	0.035
cT factor (cT1/cT2/cT3/cT4)	107/24/23/0	50/37/46/3	*p* < 0.0001	41/20/20/0	38/22/20/1	*p* = 0.90	0.173
cN factor (cN0/cN1/cN2/cN3)	94/38/18/4	34/58/36/8	*p* < 0.0001	29/32/16/4	27/34/15/5	*p* = 0.97	0.083
Preoperative therapy (Yes/No)	143/11	134/2	*p* = 0.02	79/2	79/2	*p* = 1.00	<0.001
*CF*	131	109		69	66		
*DCF*	1	5		1	5		
*CRT*	11	18		9	7		
*RT*	0	1		0	0		
*others*	0	1		0	1		
Reconstructed organ (stomach/others)	145/9	134/2	*p* = 0.07	76/5	80/1	*p* = 0.21	0.264
Anastomotic leakage (%)	2 (1.3)	4 (2.9)	*p* = 0.42	0 (0)	3 (3.7)	*p* = 0.25	0.277
Postoperative pneumonia (%)	12 (7.8)	7 (5.2)	*p* = 0.48	6 (7.4)	6 (7.4)	*p* = 1.00	<0.001
Recurrent nerve paralysis (%)	10 (6.5)	15 (11.0)	*p* = 0.21	4 (4.9)	12 (14.9)	*p* = 0.06	0.336
Clavien–Dindo grade ≥ III	20 (13.0)	24 (19.9)	*p* = 0.15	10 (12.4)	15 (18.5)	*p* = 0.38	0.13

Footnotes: CF: Cisplatin + 5FU regimen; DCF: Docetaxel + cisplatin + 5FU regimen; CRT: Chemoradiotherapy; RT: Radiation alone.

**Table 2:** Recurrence pattern among patients who developed recurrence in the two-field lymphadenectomy (2FL) and prophylactic three-field lymphadenectomy (p3FL) groups.

Recurrence Pattern	2FL Group (*n* = 154)	p3FL Group (*n* = 136)
Local	3 (1.9%)	0 (0%)
Regional LN	6 (3.9%)	18 (13.2%)
Cervical LN	4 (2.6%)	3 (2.2%)
Distant LN	2 (1.3%)	6 (4.4%)
Other organ	8 (5.2%)	7 (5.1%)
Mixed	6 (3.9%)	16 (11.8%)

Notes: LN: Lymph node.

**Table 3:** Long-term outcomes of solitary cervical lymph node recurrence cases in the two-field lymphadenectomy (2FL) group.

Case	Age	Sex	Tumor Location	cStage	Time to Recurrence (Day)	Treatment After Recurrence	Survival After Recurrence (Day)	Outcome
#1	64	Female	Mt	II	477	CRT	413	Death from primary disease
#2	72	Male	Mt	IVA	736	RT	1262	Death from primary disease
#3	61	Male	Mt	II	1090	RT	400	Death from primary disease
#4	76	Male	Mt	II	279	CRT	2585	Death from other causes

Key Abbreviations: CRT: Chemoradiotherapy; RT: Radiation alone.

## Data Availability

The datasets generated and analyzed during the current study are available from the corresponding author upon reasonable request, subject to institutional privacy regulations.

## Abbreviations

The following abbreviations are used in this manuscript:

CF	Cisplatin and 5FU
CT	Computed tomography
DCF	Docetaxel, cisplatin, and 5FU
ESCC	Esophageal squamous cell carcinoma
Mt	Middle thoracic esophagus
RCT	Randomized controlled trial
Ut	Upper thoracic esophagus
2FL	Two-field lymphadenectomy
p3FL	Prophylactic three-field lymphadenectomy
